# Community-Engaged Approach for Health Equity: Enhancing an Active Environment in Michigan

**DOI:** 10.1089/heq.2023.0237

**Published:** 2024-10-22

**Authors:** Tsu-Yin Wu, Xining Yang, Alex Swartzinski, Jessica Kwek

**Affiliations:** ^1^ Center for Health Disparities Innovations and Studies, Eastern Michigan University, Ypsilanti, Michigan, USA.; ^2^ Geography and Geology, Eastern Michigan University, Ypsilanti, Michigan, USA.

**Keywords:** health equity, physical activity, policy system and environmental (PSE) changes

## Abstract

**Introduction:**

During the COVID-19 pandemic, a decrease in physical activity (PA) has been reported in the United States and worldwide. Post-COVID-19, there is thus an urgent need for public health initiatives to revive individuals’ interest and support for regular PA. The academic-community partnership between the EMU REACH team and community stakeholders co-designed and implemented an actionable alley activation. The objectives were to (1) Apply a community-based participatory approach for conducting walk audits, and (2) Evaluate the implementation and effectiveness of the alley activation.

**Materials and Methods:**

The intervention took place in Hamtramck, Michigan and the project team engaged the stakeholders, performed environmental scans, assessed the setting, developed and implemented alley activation, and evaluated the process and outcomes of the intervention. The pilot was assessed using evaluation surveys in multiple languages and speed studies with traffic data.

**Results:**

Seventy-two residents and business owners helped implement the alley activation; 54 completed surveys offering feedback about the design and their experiences; and 1,669 residents saw immediate improvements in non-motorized connectivity. The evaluation surveys showed that respondents were positive about the project. For the traffic studies, vehicle speeds were reduced from an average of 28.3 MPH during conflicts with pedestrians to 23 MPH, and total conflicts were also reduced.

**Conclusion:**

This quick-build project served as an initial effort for the future implementation of other place-making strategies. The bottom-up community-engaged process has the potential to create a safe, appropriately scaled space that will promote increased walkability in an inner city.

## Introduction

Despite the fact that physical activity (PA) has been linked to numerous disease prevention and physical and mental well-being benefits, only 26% of men, 19% of women, and 20% of adolescents in the United States (U.S.) get the recommended daily amounts of PA.^1^ An analysis of 2020 National Health Interview Survey data showed a low proportion of U.S. adults met leisure-time aerobic, muscle-strengthening, and combined PA guidelines while residents in the Midwest U.S. Census Bureau region were less likely than average to meet these guidelines.^2^ Racial/Ethnic minorities and those from socioeconomically disadvantaged communities are even less likely to engage in sufficient PA.^1,3,4^

A well-designed built environment that promotes PA reduces physical inactivity levels and promotes public health. The Community Preventive Services Task Force showed combinations of activity-friendly built environment characteristics are associated with higher levels of transportation-related physical activity, recreational physical activity, and total walking.^5^ A review of open space and street connectivity and their relationships with PA at a population level showed that open space and street connectivity have a beneficial effect on PA.^6^ Enhancing the built environment can thus be an effective tool for improving PA levels among disadvantaged populations.^7^

The Healthy People 2030 objective PA-10 is to increase the proportion of adults who walk or bike to get places with the target goal of 26,8% whereas at the baseline, 22.4% of adults aged 20 years and over walked or used a bicycle to get to and from places in 2013–2016 and the percentage decreased to 21.6% in 2017–2020.^8^ A decrease in physical activity levels has been reported in other countries during the COVID-19 pandemic.^9^ There is an urgent need for public health initiatives to revive individuals’ interest and support for performing regular PA post-COVID-19.

Policy, systems, and environmental (PSE) change approaches are a multi-sector endeavor to promote sustainable opportunities for promoting PA within communities.^10^ Using community engagement ensures leveraging knowledge of local needs, and empowering communities within a defined geographic area,^11^ ultimately, creating effective solutions tailored to the needs of affected communities. Alleys can contribute around 50% of additional public space to the city creating a new network for pedestrians.^12^ Community-engaged alley activation is a strategy to promote physical activity and it can transform neglected spaces into vibrant community hubs and foster social interaction and community cohesion.^13^

## Purpose and objectives

The project team at Eastern Michigan University (EMU) received five-year support from the Centers for Disease Control and Prevention (CDC) Racial and Ethnic Approaches to Community Health (REACH) cooperative agreement to implement sustainable PSE change strategies in addressing the chronic disease burden among under-resourced economically disadvantaged communities in Michigan with priority populations of Asian Americans. This case study applied the Community Guide’s environmental approaches that create supportive conditions for individuals to adopt PA. The EMU project team implemented PSE change strategies combined with principles of the community-based participatory approach: Engaging community members and building partnerships, performing environmental scans, identifying priority areas and assessing the setting, developing and implementing the PSE intervention, and evaluating the process and outcomes of the intervention.

The purpose of this article is to describe an academic-community partnership between the EMU REACH team and community stakeholders to advance health equity and promote an active environment. The objectives were to (1) Perform environment scans with community-engagement approaches in walk audits, and (2) Evaluate the implementation and effectiveness of a community-engaged alley activation.

## Project site

Hamtramck is Michigan’s most densely populated city, with over 28,000 people residing in a city that spans just 2.1 square miles. This small city is bordered on most sides by Detroit, Michigan’s largest city, and by Highland Park on one side. Partly because of this proximity to a historically industrial city, Hamtramck has been home to immigrants from Poland, Ukraine, and other Eastern European countries settling in the city beginning in the early 1900s. In recent years, the city has seen an influx of immigrants from countries such as Bangladesh and Yemen. As of 2020, 40% of Hamtramck’s residents are foreign-born, immigrating from countries in Eastern Europe, South Asia, and the Middle East.^14^ Hamtramck residents also experience high poverty rates of 43.1%.^15^

Despite Hamtramck’s dense and walkable nature, residents avoid walking or biking in the city because of obstacles such as wide streets with minimal crosswalks, deteriorating or incomplete infrastructure, unsafe conditions created by speeding or negligent drivers, and a lack of knowledge in the community about safe interactions with pedestrians or cyclists. About one-third of Hamtramck residents do not drive, and 10% do not have access to a vehicle, making some of the city’s most vulnerable residents subject to increased danger out of necessity.^16^

The Joe Louis Greenway project is planned to span 27.5 miles through Detroit, Hamtramck, Highland Park, and Dearborn. The City of Hamtramck initially created a plan for protected, two-way bike lanes along Joseph Campau Street, the city’s main thoroughfare. It was later determined that this section of the Joe Louis Greenway in Hamtramck was too narrow to include on-street bike lanes and the alley behind businesses on the west side of Joseph Campau was deemed as an alternate route of the greenway.

## Community engagement

The Asian Communities toward Innovative Visionary Environment (ACTIVE) Coalition, a state-wide coalition with the mission of promoting the health and well-being of underserved disadvantaged communities, tasked a subset of coalition members to develop and conduct a needs assessment to promote PA. Members of this workgroup included the EMU PA team, community members, business owners, the city beautification commission, the city of Hamtramck, and students from local schools. The workgroup conducted an environmental scan to identify gaps, trends, and factors affecting the local environment contexts to understand the drivers of PSE changes for PA and determine next-step actions.

## Environmental scans

Environmental scanning and walk audits have been widely reported as critical and effective tools in active transportation planning and implementation. In the summer of 2020, the project team conducted walk audits to assess the walkability of the environment and identify opportunities for creating a more active environment. The initial planning included creating a walk audit tool adapted from AARP^17^ and a companion YouTube tutorial,^18^ for using the toolkit. The walk audit toolkit provides a framework to observe and evaluate the walkability of a location and consists of step-by-step instructions and checklists for examining intersections, sidewalks, driver behavior, public safety, and the comfort of the street. Due to COVID-19 restrictions and to comply with social distancing guidelines, six community volunteers (residents of the neighborhood, owners/employees of local businesses interested in improving the walkability of their community) completed walk audits on 12 routes with a total length of 8 miles in the city. A total of 205 photos were taken; a Geographic Information System (GIS) software ESRI ArcGIS Pro,^19^ was used to geocode the photos and digitize the qualitative comments associated with the photos. An interactive web-based dashboard,^20^ visualized the result of environmental scanning and disseminated it to the community.

**FIG. 1.**
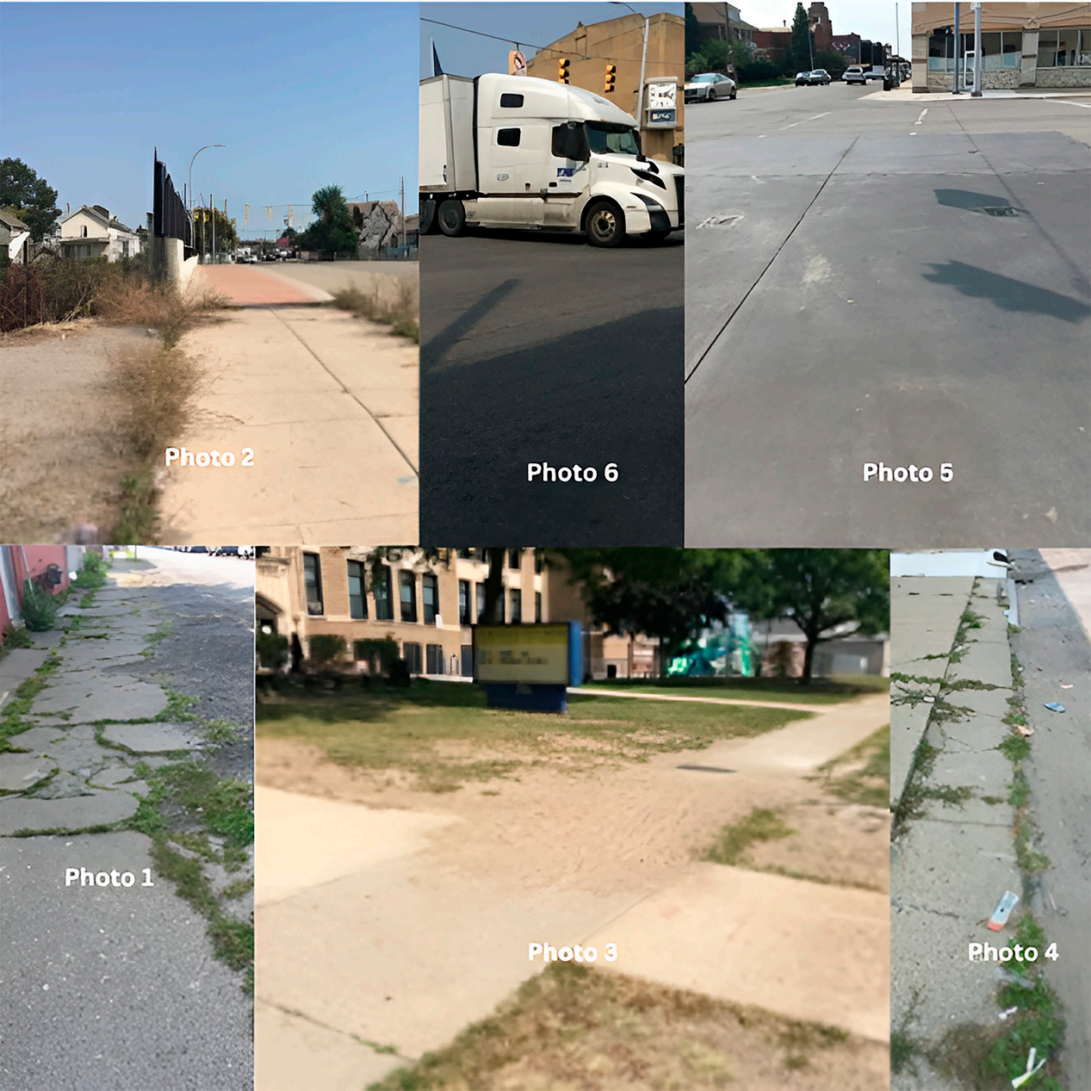
Current pedestrian infrastructure conditions in Hamtramck.

After the walk audit, 10 community residents were invited to join a virtual online focus group (via Zoom) and asked to review 6 photos (Fig. 1) and respond to 5 questions: What do you see here? What is really happening here? How does this relate to our lives? Why does this problem, concern, or strength exist? What can we do about it?

After the online focus group, the walk audit results were coded into Opportunities, Challenges, and Barriers and a report has been shared with the stakeholders including the city of Hamtramck and the Joe Louis Greenway team. The interactive web-based dashboard was used by the city Department of Public Work to identify high-risk areas for sidewalk repairs and maintenance.

## Walk audit findings and recommendations

Overall, participants expressed a need for a safe, clean neighborhood that cultivates a sense of community and civic space, celebrates Hamtramck’s rich history and diversity, and supports local businesses that invest in the community. General themes from the recommendations included:

- *Cleaning up the alley and creating a clean and safe environment that promotes walkability*

Quotations (responding to Fig. 1: photo 4) were “…just felt bad for the environment because there was so much trash, like paper and bottles thrown out,” “...is cleanliness being associated with safety…” Most of the participants made the association that these streets aren’t safe to walk on.

Quotations (responding to Fig. 1: photo 2): “…have walked over this bridge before and neither of us felt very comfortable being in this area.” Another quotation showed safety concerns for younger residents, “Kids use this to walk to school sometimes, during the winter month, when the sun comes out a little bit later…and our top priority is to know **how safe it is** for those children (to) walk to school.”

- *Drivers’ speeding behavior at intersections makes pedestrian and non-motorized transportation a challenge to cross the intersection. Traffic calming is needed.*

Quotation (responding to Fig. 1: photo 6) reflected speeding is a major concern, “I stood at this intersection,… **At least two of them were speeding,** one of them ran a yellow light and turned, and there were pedestrians in the area. I saw people crossing this intersection with walkers, bikes, people who had challenges getting across this intersection.”

- *Finding solutions for traffic calming in the Hamtramck Downtown Development Authority (DDA) area, where the Joe Louis Greenway Hamtramck segment was located.*

Quotations (responding to Fig. 1: photo 5) provided potential actionable items “...(to) increase the walkability, it would certainly **slow down the cars,** and it would make it a lot easier to cross the street on the rest of Joseph Campau in the DDA area.” Another quotation lent support for the actionable point, “…it also draws drivers’ eyes to that area a little bit more so they can potentially pay more attention and see pedestrians in that area.”

## Engaging the community in building local greenway

The EMU-REACH PA team worked with a Joe Louis Greenway Hamtramck Community Coalition (JGHCC) consisting of business owners, students, and residents and formed a Quick Build Team. They developed a plan for an alternative route that would activate an alley behind businesses on the west side of this section of Joseph Campau and create a shared space for pedestrians, slow bicyclists, and businesses. To garner more support from community members, the JGHCC’s Quick Build Team organized a three-day alley activation event where residents helped to build planter box bump-outs for traffic calming, paint markings in the alley to designate use and build seating in the alley to create a safe and inviting space for pedestrians and cyclists. The city also commissioned a local artist to paint a mural in the alley commemorating the Greenway’s namesake, Joe Louis, and create visual interest.

The alley activation event on November 6, 2021, allowed City of Hamtramck staff and volunteers to answer questions, hear feedback, and address concerns. Community participants learned more about the vision behind the design and how it would impact them. As a result, 72 residents and business owners helped to implement the alley activation; 54 completed surveys offering feedback about the design and their experience; and 1,669 residents saw immediate improvements in non-motorized connectivity.

### Project evaluation

The project team utilized mixed methods to collect data about this alley activation project. The evaluation had two components: (1) A 15-item evaluation survey and (2) Speed studies with traffic data.

The evaluation survey included demographics (Table 1), transportation behaviors and experience levels with pedestrian and bicycle modes of active transportation, safety perception with these modes of transportation, prior knowledge of the Joe Louis Greenway, and opinions regarding the proposed alley design. The questions involve step-by-step inquiring about their experience of different transportation modes including physical activity and walking and their perceptions about safety with the particular type of transportation. The safety and accessibility of built environments directly influence transportation decisions and community health outcomes.^8^ Studying transportation modes within Hamtramck is crucial to understanding this relationship between access and equity. The surveys were distributed during community events in the City of Hamtramck and available in Arabic, Bengali, and English.

**Table 1.** Evaluation Survey Demographics

Gender identity (%)	
Male	48
Female	52
Another gender identity	0
Race or Ethnicity (%)	
Asian American	34
White	18
Black	8
Yemi	3
Bengali	3
Not answered	34
Age (%)	
Under 18	16
Between 18–30	13
Between 31–50	26
Between 51–70	15
Greater than 71	4
Answered “Senior”	2
Not provided	24

Traffic data was collected by two resident volunteers on the streets of Trowbridge and Belmont, where the traffic calming intervention was implemented. Speed limits on Michigan residential roadways are 25 MPH unless posted otherwise.^21^ Data were collected in 5 min intervals at three different times of the day classified as “morning, noon, and afternoon “with three data points: before alley activation (11/05/2021), during the alley activation (11/06/2021), and after the alley event had concluded (11/07/2021). Trained volunteers collected speed data using Bushnell Velocity Value Kit Bushnell 101,911 Radar Guns and also noted unsafe interactions between pedestrians and motor vehicle traffic whereas two categories were used with “conflict” vs. “no conflicts” in the visualized datasets.

## Results

### Survey data

Among 54 surveys completed, (52% were females. Respondents were diverse, and Asian Americans composed 34.3% of all respondents followed by White residents (18.8%), Black residents (8.3%), Yemeni and Bengali residents (2.8%), respectively. Safety patterns and concerns about active transportation (walking and biking) were reported by participants; 77% rode a bike and more than 90% often and sometimes walked, 52% felt unsafe about biking, and 7% felt unsafe about walking in Hamtramck. About 45% of participants had not heard of the Joe Louis Greenway project.

The survey showed a great deal of interest in the space both by people who came intentionally to see the installation and by those who just happened to pass by. Some quotations from the surveys are as follows:

“It would be so amazing to have an area like this where you can relax outside with friends and family in the city.”

“If it is like this (pop-up) it will be amazing for people to walk, and it will be safe and attractive.”

“Awesome work! Because Hamtramck is so packed together, even a few spaces like this go a long way to encourage people to spend more time in the community/community spaces.”

“Sometimes kids play cricket and other games here. Would be cool to have painted lines for hopscotch, cricket, etc. Please push for mirrors, speed bumps, etc.!”

“This is a commendable undertaking. It is welcome.”

“Neat and clean and safe walking.”

**FIG. 2.**
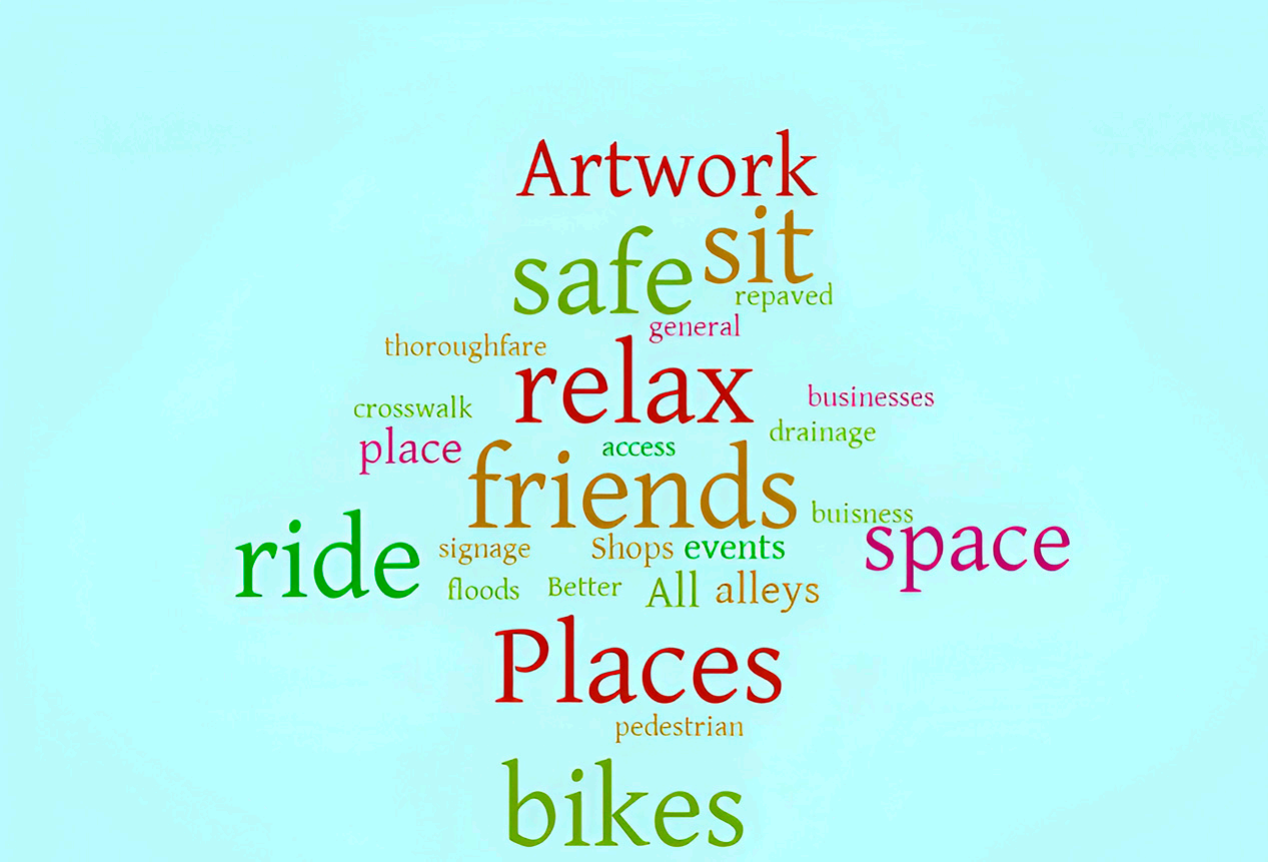
Community member answers to the prompt: “What would you like to see in the alley most?”.

To reveal relevant themes and trends, a word cloud (Fig. 2) has been created to analyze the survey responses to the question, “What would you like to see in the alley most?” One common theme is safety as some respondents expressed the need to improve the alley design for a safe environment including ideas to add a crosswalk and repave the surface. The economic benefit is another common theme worth noting including the desire to add shops, benches, and signage which help to draw foot and bike traffic to nearby businesses, boosting local economies.

### Traffic data

**FIG. 3.**
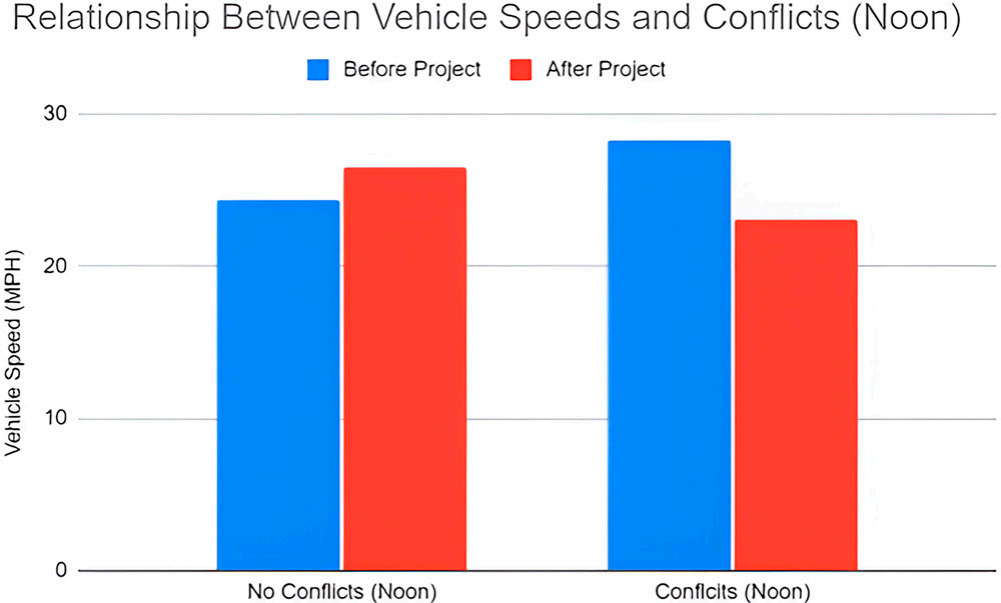
Vehicle mean speeds at noon.

**FIG. 4.**
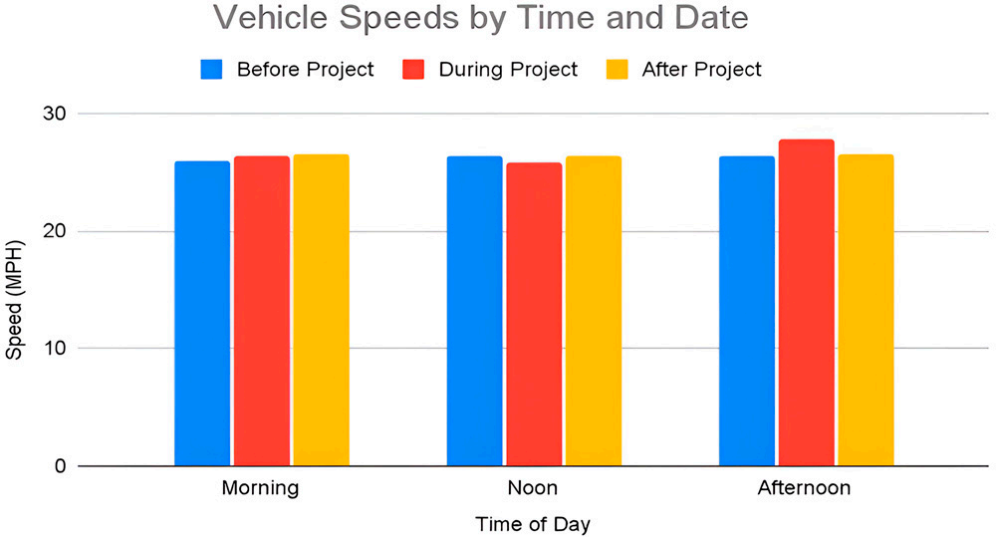
Vehicle speeds collected by time and date before, during, and after the quick build event.

**FIG. 5.**
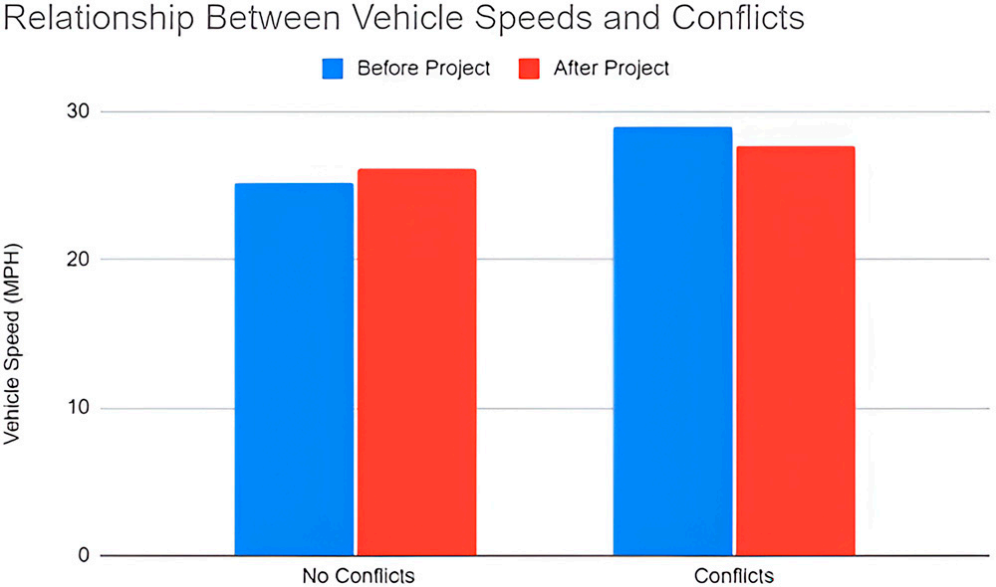
Relationship between vehicle speeds and pedestrian conflict interactions.

Using radar data, we calculated the average roadway conditions which resulted in dangerous conflicts between motor vehicles and pedestrians. The mean speeds during the event at noon were slower than before and after the event (Fig. 3). There was little difference in remaining speeds at various times of the day (Fig. 4). In terms of speeds with dangerous conflicts between vehicles and pedestrians, the mean speed was 28.9 MPH (Fig. 5) while the time of day affected these results. Vehicle speeds were highest during the early afternoon in 41% of conflict interactions. Lower vehicle speeds result in fewer conflicts. Of the 58 interactions that did not result in a dangerous interaction, the mean speed was 25.7 MPH. The early morning hours had the fewest conflicts and the lowest vehicle speeds whereas early morning speed measurements composed 34% of the dataset, they only recorded 16% of all conflicts.

## Discussion

This article described a community-engaged approach to involve residents from vulnerable communities in a quick-build alley activation, a small-scale PSE-level initiative within the setting of an impoverished neighborhood, in Hamtramck, Michigan. Community-based participatory research principles including promoting co-learning and capacity-building among partners, shared decision-making, and mutual ownership formed the foundation of project strategies. This academic-community partnership ensured community participation in all aspects during the project, including forming a workgroup to guide project design; performing an environmental scan with walk audits, implementing and evaluating alley activation, and collecting the process and traffic data. These strategies bridge the gap between research and practice through the equitable engagement of the community to eliminate disparities in population health.^22,23^

New lessons emerged from the current project. First, the COVID-19 pandemic forced the project team to pivot; walk audits with virtual focus group discussions were shown to be feasible and opened doors to include a broader pool of participants with flexibility of location and scheduling. Previous studies documented that marginalized urban communities do not have access to the economic and formal infrastructure of the city.^24,25^ Similarly in the current project, almost half of respondents reported they did not know about the major city transportation improvement, the Joe Louis Greenway project, which may be due to their lived experiences or cultural and language barriers. Involving community members in a series of quick-build activities promotes citizen involvement and decision-making in local-government-led initiatives.

Historically, alleys are a common form of urban infrastructure with the purpose of trash collection, alternative entry for residences, or storage of motor vehicles. The City of Hamtramck stopped maintaining alleys in the late 1980s, passing control to homeowners and relying on residents to maintain the upkeep of the alleys. As a result, many alleys became unattended and residents expressed safety concerns and littering issues. The small-scale project described in this article encouraged bottom-up community engagement that empowered community residents to plan, develop, and implement a local urban intervention. This is essential for long-term sustainability as it can be difficult to maintain support without local buy-in and solutions from the community itself for a resource-limited city such as Hamtramck.

The project team recognized through surveys and coalition meetings with residents that the vast majority of community members still express safety concerns. Infrastructure has historically favored automobile drivers; wide streets promote high vehicle speeds which are unsafe for active transportation users.^26^ Inconsistent sidewalk and crosswalk placement create dangerous conflicts between high-speed automobile traffic and vulnerable active users. The alley activation project demonstrated that reducing vehicle speeds through community outreach and infrastructure improvements can promote public safety. As established before, there is a known correlation between speeds and injury rates. According to Tefft,^27^ serious injuries to the pedestrian occurred in 25% of cases with a vehicle traveling at 23 MPH in a vehicle-pedestrian collision; when speeds were increased to 31 MPH, the risk of serious injury rose to 50%. Small-scale outreach projects like this one have the potential to decrease vehicle speeds while promoting safe biking and walking practices. This alley activation demonstrated lowered vehicle speeds with temporary low-cost infrastructure improvements, and total conflicts were also reduced. More data points in future traffic studies can further validate the findings.

While initial evidence from the survey suggests that environment-level intervention is acceptable to the community residents and business owners, the survey data is limited with small sample size and inability to administer the pre-and post-surveys to assess change in the built-environment intervention. The project team is currently working on the Joe Louis Greenway implementation with the City of Hamtramck and community partners. We plan to conduct surveys and interviews with key stakeholders (i.e., residents, business owners, and city staff) and obtain information regarding facilitators and barriers to the sustainability of the intervention.

The current project demonstrated the process of engaging residents from under-resourced low-income communities with a systematic tool that they can apply in bringing about positive local change. Empowering residents to learn about how to change elements of their local environments and later using the results to work with their city’s planning and engineering divisions to improve neighborhood conditions has implications for potentially advancing the goal of health equity for all. Future studies can further expand the evaluation of the impacts of the intervention on multi-level, for example, individual- and neighborhood-level outcomes, and identify strategies for sustainability of the community-engagement process to other relevant health-related areas and communities.

The quick-build project serves as a starting point for the implementation of alley activation and other place-making strategies in other locations along the Joe Louis Greenway. The community-engaged process has the potential to create a safe, appropriately scaled pedestrian network that will promote walkability in an inner city. The success of this pilot project also calls for future replication and adoption of PSE approaches to promote PA through a community-driven design.

## Abbreviations Used

AARP: American Association of Retired Persons
ACTIVE: Asian Communities toward Innovative Visionary Environment
CDC: Centers for Disease Control and Prevention
CHDIS: Center for Health Disparities Innovations & Studies
DNPAO: Division of Nutrition, Physical Activity, and Obesity
EMU: Eastern Michigan University
GIS: Geographic Information System
JGHCC: Joe Louis Greenway Hamtramck Community Coalition
MPH: Miles per hour
PA: Physical Activity
PSE: Policy, systems, and environmental
REACH: Racial and Ethnic Approaches to Community Health
U.S.: United States of America

## References

[1] U.S. Department of Health and Human Services. Physical Activity Guidelines for Americans 2nd Edition. 2018. Available from: https://health.gov/sites/default/files/2019-09/Physical_Activity_Guidelines_2nd_edition.pdf [Last accessed: October 21, 2023].

[2] Abildso CG, Daily SM, Umstattd Meyer MR, et al. Prevalence of meeting aerobic, muscle-strengthening, and combined physical activity guidelines during leisure time among adults, by rural-urban classification and region—United States, 2020. MMWR Morb Mortal Wkly Rep 2023;72(4):85–89; doi: 10.15585/mmwr.mm7204a1 36701252 PMC9925130

[3] Conn VS, Coon Sells TG. Effectiveness of interventions to increase physical activity among minority populations: An umbrella review. J Natl Med Assoc 2016;108(1):54–68; doi: 10.1016/j.jnma.2015.12.008 26928489

[4] Cooper J, Stetson B, Bonner J, et al. Self-reported physical activity in medically underserved adults with Type 2 diabetes in clinical and community settings. J Phys Act Health 2015;12(7):968–975; doi: 10.1123/jpah.2013-0475 25154022

[5] Physical Activity: Built Environment Approaches | The Community Guide. www.thecommunityguide.org; 2022. Available from: https://www.thecommunityguide.org/findings/physical-activity-built-environment-approaches.html [Last accessed: October 21, 2023].

[6] Pearce JR, Maddison R. Do enhancements to the urban built environment improve physical activity levels among socially disadvantaged populations? Int J Equity Health 2011;10(1):28; doi: 10.1186/1475-9276-10-28 21767365 PMC3161844

[7] Christie CD, Consoli A, Ronksley PE, et al. Associations between the built environment and physical activity among adults with low socio-economic status in Canada: A systematic review. Can J Public Health 2020;112(1):152–165; doi: 10.17269/s41997-020-00364-9 32833139 PMC7851286

[8] Office of Disease Prevention and Health Promotion. Social Determinants of Health. Healthy People 2030. U.S. Department of Health and Human Services. (n.d.). Available from: https://health.gov/healthypeople/priority-areas/social-determinants-health [Last accessed: October 21, 2023].

[9] Wilke J, Mohr L, Tenforde AS, et al. A Pandemic within the Pandemic? Physical Activity Levels Substantially Decreased in Countries Affected by COVID-19. Int J Environ Res Public Health 2021;18(5):2235; doi: 10.3390/ijerph18052235 33668262 PMC7967678

[10] Lyn R, Aytur S, Davis TA, et al. Policy, Systems, and Environmental Approaches for Obesity Prevention. J Public Health Manag Pract 2013;19(3 Suppl 1):S23–S33; doi: 10.1097/phh.0b013e3182841709 23529052 PMC4943076

[11] Goins KV, Schneider KL, Brownson R, et al. Municipal officials’ perceived barriers to consideration of physical activity in community design decision making. J Public Health Manag Pract: JPHMP 2013;19(3 Suppl 1):S65–S73; doi: 10.1097/PHH.0b013e318284970e PMC4928376 23529058

[12] Ink S. Seattle Integrated Alley Handbook: Activating alleys for a lively city. National Association of City Transportation Officials; 2015. Available from: https://nacto.org/references/fialko-mary-and-jennifer-hampton/

[13] Peterson E. Alley activation: Pathways to urban revitalization. TheLineMedia; 2014. Available from: https://www.thelinemedia.com/features/alleyactivation04232014.aspx

[14] Kaymond K. One small city, three vibrant immigrant communities. Michigan Radio; 2019. Available from: https://www.michiganradio.org/families-community/2019-10-15/one-small-city-three-vibrant-immigrant-communities [Last accessed: June 29, 2023].

[15] U.S. Census Bureau QuickFacts: Hamtramck city, Michigan. www.census.gov; (n.d.). Available from: https://www.census.gov/quickfacts/fact/table/hamtramckcitymichigan [Last accessed: June 29, 2023].

[16] Explore Census Data. data.census.gov. 2020. Available from: https://data.census.gov/table?q=Hamtramck+city [Last accessed: June 29, 2023].

[17] Communities AL. AARP Walk Audit Tool Kit. AARP. 2022. Available from: https://www.aarp.org/livable-communities/getting-around/aarp-walk-audit-tool-kit.html [Last accessed: June 29, 2023].

[18] Donnelly J. EMU REACH Project: Completing a Walk Audit Using the AARP Walk Audit Tool Kit. www.youtube.com. 2020. Available from: https://youtu.be/XLOONWLsEb8 [Last accessed: June 29, 2023].

[19] ArcGIS Pro | 2D and 3D GIS Mapping Software. Esri.com. 2019. Available from: https://www.esri.com/en-us/arcgis/products/arcgis-pro/overview [Last accessed: June 29, 2023].

[20] EMU CHDIS Virtual Walk Audit Project Dashboard. 2021. Available from: https://igre.maps.arcgis.com/apps/dashboards/b26aa06e89e54a6c9a9818371e589341 [Last accessed: June 29, 2023].

[21] Michigan Legislature - Section 257.627. www.legislature.mi.gov. Available from: http://www.legislature.mi.gov/(S(4phhxqerhdoahvc22z4imv5f))/mileg.aspx?page=GetObject&objectname=mcl-257-627 [Last accessed: June 29, 2023].

[22] Traffic Monitoring Program. Michigan.gov; 2023. Available from: https://www.michigan.gov/mdot/programs/planning/asset-mgt/traffic-monitoring-program; [Last accessed: June 29, 2023].

[23] Wallerstein N, Duran B. Community-based participatory research contributions to intervention research: The intersection of science and practice to improve health equity. Am J Public Health 2010;100(Suppl 1):S40–S46; doi: 10.2105/ajph.2009.184036 20147663 PMC2837458

[24] Cahyani D, Widaningsih L. Identification of the marginalized urban communities characteristics and preferences. KnE Soc. Sci 2019;3:178–192; doi: 10.18502/kss.v3i21.4967

[25] Williams DS, Balaban O, Ilhan A, et al. A policy content analysis for evaluating urban adaptation justice in İstanbul. Environ Sci. Policy 2022;136:476–485; doi: 10.1016/j.envsci.2022.07.014

[26] Salerno C. Pedestrian Safety Archives. Transportation for America. 2010. Available from: https://t4america.org/tag/pedestrian-safety/ [Last accessed: October 21, 2023].

[27] Tefft B. Impact Speed and a Pedestrian’s Risk of Severe Injury or Death. AAA Foundation; 2011. Available from: https://aaafoundation.org/impact-speed-pedestrians-risk-severe-injury-death [Last accessed: October 21, 2023]. 10.1016/j.aap.2012.07.022 22935347

